# Everybody's Page

**Published:** 1891-10-31

**Authors:** 


### EVERYBODY'S PAGE.

IT is asserted that lard may be made perfectly sweet by boiling a pared potato in it.

---

THE custom of apothecaries offering and paying physicians a percentage on their prescriptions is asserted to be scandalously prevalent in San Francisco. It is probably not unknown in other countries besides America, but the practice seems to be unpleasantly prevalent in the States.

---

A PASSENGER by one of the New York liners states that so rough has been the weather that many of his fellow passengers were unable to take their regular meals, but were comforted and supported by the Liebig Company's Extract of Meat, which, owing to its being so easily digestible and soothing in its effect on the nerves of the stomach, they were able to take when other food was nauseating to them.

---

ONE section of the British Columbia Pharmacy Act entitles any person presenting a prescription to a chemist to have it returned to him by the chemist. This practically defines the ownership of the prescription, which has been a debated question. Little harm is done probably by the circulation of some prescriptions, but in many cases infinite harm might be done by allowing a prescription to become common property. Some physicians have a wise practice of tearing up a prescription they have written for a patient when they consider it no longer required, but we presume they could not take this step if the ownership were decided to rest in the patient.

---

MANY people who have been prescribed iron have found it inimical to the digestion. Essential oil of thyme, made into a pill with soap and althæa, is asserted to aid its digestion.

---

SIMPLE remedies are often the best, but we cannot vouch for the efficacy of pineapple juice in diphtheria, or of banana juice in cases of chronic bronchitis with difficult breathing and scanty expectoration. As a syrup, each doubtless has soothing qualities, but beyond that their value must be questionable.

---

THERE appears to be a splendid field for quacks in Australia. One of these fortunate gentlemen is stated to have admitted to a Royal Commission that he had amassed £100,000 by his practice. One cause of this state of affairs is that in the colonies the tie between doctor and patients is very loose. Colonial life is of a restless character, and patients seldom remain long under one physician. Thus occurs an opening for the quack, of which he is not slow to avail himself.

---

A GENTLEMAN who was of a thrifty, not to say mean, turn of mind, was anxious to get medical advice gratis. He therefore managed to meet his physician while on his rounds—of course just by accident. He addressed him: "How are you, you're looking wonderfully well. If you had a constant tickling in your throat like I have, I bet you wouldn't be looking so fit. What do you generally do when you have a tickling in your throat?" "I generally cough," replied the Doctor, passing on.

